# Cavity expansion theory with state-dependent mohr-coulomb model and its application to cone penetration tests

**DOI:** 10.1371/journal.pone.0329935

**Published:** 2025-08-20

**Authors:** Baojian Li, Sai Fu, Xiaoyu Feng, Jian Shen, Bing Duan, Li Pang

**Affiliations:** 1 PowerChina Huadong Engineering Corporation Limited, Hangzhou, China; 2 Zhejiang Institute of Communications Co. Ltd., Hangzhou, Zhejiang, China; 3 College of Civil Engineering and Architecture, Zhejiang University, Hangzhou, China; China Construction Fourth Engineering Division Corp. Ltd, CHINA

## Abstract

The cone penetration test (CPT) is a fast and efficient in-situ testing technique that provides reliable and continuous measurements of soil properties. The CPT calibration chamber test is widely used to investigate soil-pile interactions. To address the boundary effect problem in CPT calibration chamber tests, this paper applies the cavity expansion theory for analysis. In this approach, the stress-strain relationship of soil is modeled using the classical Mohr-Coulomb (M-C) model, where the elastic modulus is associated with the mean stress, and the internal friction angle and dilation angle are related to the void ratio and mean stress. This modification captures the state-dependent characteristics of sand. By combining the stress equilibrium equation and the volume conservation equation, a system of partial differential equations (PDEs) is established to describe the stress-strain behavior of the soil element. The hybrid Eulerian-Lagrangian approach is employed to solve these PDEs, yielding the pressure-expansion curve and the stress distribution curve along the cavity wall during the expansion process of the cylindrical (spherical) cavity. The results of this semi-analytical solution are compared with the exact solution to validate the accuracy of the proposed method. Additionally, the relationship between the cylindrical (spherical) cavity expansion model and the cone penetration resistance in CPT is established. The development curve of critical depth with cone penetration resistance is accurately predicted.

## 1. Introduction

Cone penetration test (CPT) is an in-situ testing technique with a wide range of applications and high data quality, which can compensate for the shortcomings of laboratory geotechnical tests [1]. The calibration chamber test for CPT is an effective means to verify the accuracy of CPT testing and inversion. How to determine the size of the calibration chamber is an important issue in CPT [2]: When the size of the calibration chamber is too small, the boundary effect of the calibration chamber has a certain impact on the measured cone penetration resistance [3,4]; When the size of the calibration chamber is too large, it will reduce experimental efficiency and undertake more resources.

The research on the theory of cone penetration test (CPT) mainly includes the bearing capacity method, motion point displacement method, strain path method, and cavity expansion theory [5]. Since Vesic [6] proposed the theory of cavity expansion theory, it has been widely applied in fields such as in-situ testing, pile foundations, and tunnel excavation. This theory can currently be roughly divided into two methods: the ‘Eulerian method’ and ‘Lagrangian method’. The Eulerian method highly relies on the self-similarity characteristic of the cavity expansion process, that is, all soil elements around the cavity experience the same stress-strain path; The Lagrangian method can be used to solve both self-similar and non-self-similar problems. This method usually requires an analytical form between stress and total strain, so the used constitutive model is relatively simple. Recently, Yang et al. [7–9] proposed a hybrid Eulerian-Lagrangian (HEL) method that combines both advantages of methods, and uses a semi-numerical solution to provide a solution framework for non-self-similar cavity expansion problems in soils with sophisticated constitutive models. The authors will also use the HEL method to solve the cavity expansion problem in this paper.

Due to the relatively easy analysis of the theory of cavity expansion theory in infinite medium, the development of the cavity expansion theory in finite medium is relatively lagging behind. Therefore, researchers often use the approximate solution of cavity expansion theory to predict cone penetration resistance of CPT. Osinov and Cudamni [10] proposed a cavity expansion solution in a finite medium with a hypoplastic constitutive model, but when applied to predict cone penetration resistance of CPT, only the infinite medium case was discussed [11]. Pournaghiazar et al. analyzed the boundary effect of CPT using the cavity expansion theory based on the critical state constitutive model [12], but the solution based on the Eulerian method, which is only an approximate solution and cannot reflect of the non-self-similar behavior during the CPT process of the calibration chamber [13]. The Mohr-Coulomb (M-C) yield criterion is commonly used to analyze the size effect of calibration chamber [14–16], but the M-C model is an ideal elastoplastic model and cannot well reflect the dependence of soil strength and stiffness on stress-strain levels during CPT. Therefore, adopting a relatively simple but soil-state-related model and providing a theoretical framework for calculating the problem of cavity expansion in finite medium, can enrich the theory of cavity expansion theory and improve its applicability and accuracy in CPT prediction.

This paper proposes a solution for the cavity expansion problem in finite medium based on the state-dependent Mohr-Coulomb (SDMC) model. The elastic modulus in the classical M-C model is correlated with the mean stress, and the internal friction angle and dilation angle are correlated with the void ratio and mean stress of the soil, capturing the state-related characteristics of sand. Subsequently, combining stress equilibrium equations, displacement compatibility condition, volume conservation equation, and constitutive equation, a set of partial differential equations (PDEs) for the expansion process of cylindrical (spherical) cavity are obtained. The hybrid Eulerian-Lagrangian (HEL) method is used to solve the equations, and the pressure-expansion curve and stress distribution curve of the cavity in finite medium during expansion are obtained. The solution results obtained in this paper are compared with the exact solution results and numerical simulation results. The method established in this paper is also applied to predict the development of cone penetration resistance at critical depth during CPT penetration, verifying the accuracy of the method proposed in this paper

## 2. State-dependent Mohr-Coulomb (SDMC) model

This paper adopts a relatively simple M-C constitutive model, but associates it with state parameters to establish a state-dependent Mohr-Coulomb (SDMC) constitutive model. The model was found to give satisfactory simulations for large-deformations. The model also provided the key parameters which were made state-dependent and were calibrated carefully against high-quality element tests conducted over appropriate pressure ranges [17,18]. On the basis of the classical M-C model, this model associates the elastic modulus, internal friction angle, and dilation angle with state parameters.

The M-C model can be expressed with the maximum ($\sigma_{1}$) and minimum ($\sigma_{3}$) principal stresses as:


$$f=\sigma_{1}-\alpha \sigma_{3}-Y$$ (1)

where α=(1+sinϕ)/(1−sinϕ); Y=2ccosϕ/(1−sinϕ); ϕ is the internal friction angle of the soil; c is the cohesion force of the soil.

The M-C model adopts the non-associated flow rule, and plastic strain increments are correlated by dilation angle ($\phi_{d}$), as:


$$\frac{\text{d}\varepsilon_{1}^{p}}{\text{d}\varepsilon_{3}^{p}}=-\frac{1-\text{sin}\phi_{d}}{1+\text{sin}\phi_{d}}$$ (2)

where $\text{d}\varepsilon_{1}^{\text{p}}$ and $\text{d}\varepsilon_{3}^{\text{p}}$ are plastic strain increments corresponding to the maximum and minimum principal stresses, respectively.

The SDMC model represents the elastic modulus (E) as a function of the mean stress (p) as follows:


$$E=E_{i}p_{\text{atm}}{\left(\frac{p}{p_{atm}}\right)}^{0.5}$$ (3)

where $E_{i}$ is a constant; $p_{\text{atm}}$ is the atmospheric pressure, taken as 101 kPa.

The relationship between the internal friction angle (ϕ) and dilation angle ($\phi_{d}$) of soil and the state parameter (ψ) is described in the exponential form, as:


$$\text{tan}\phi =\text{tan}\phi_{c}exp(-n^{b}\psi )$$ (4a)

$$\text{tan}\phi_{d}=exp(-n^{d}\psi )$$ (4b)

where $\phi_{c}$ is the friction angle at the critical state; $n^{b}$ and $n^{d}$ are model constants.

The state parameter (ψ) in Eqs (4a) and (4b) defines the relationship between the current void ratio (e) and the void ratio at the critical state ($e_{c}$) of the soil, as [19]:


$$\psi =e-e_{c}$$ (5)

Li and Wang [20] expressed the void ratio of the soil at the critical state as a function of mean stress, which is:


$$e_{c}=e_{c0}-\lambda_{c}{\left(\frac{p}{p_{\text{atm}}}\right)}^{\xi }$$ (6)

and Eq (7) defines the liner relationship between $e_{c}$ and ${\left(p/p_{\text{atm}}\right)}^{\xi }$, which is also called ‘critical-state line’, where $e_{c}$ and $\lambda_{c}$ are intercept and slope of the critical-state line, respectively.

## 3. The establishment of the cavity expansion model

### 3.1 Basic assumption

**Fig 1:**
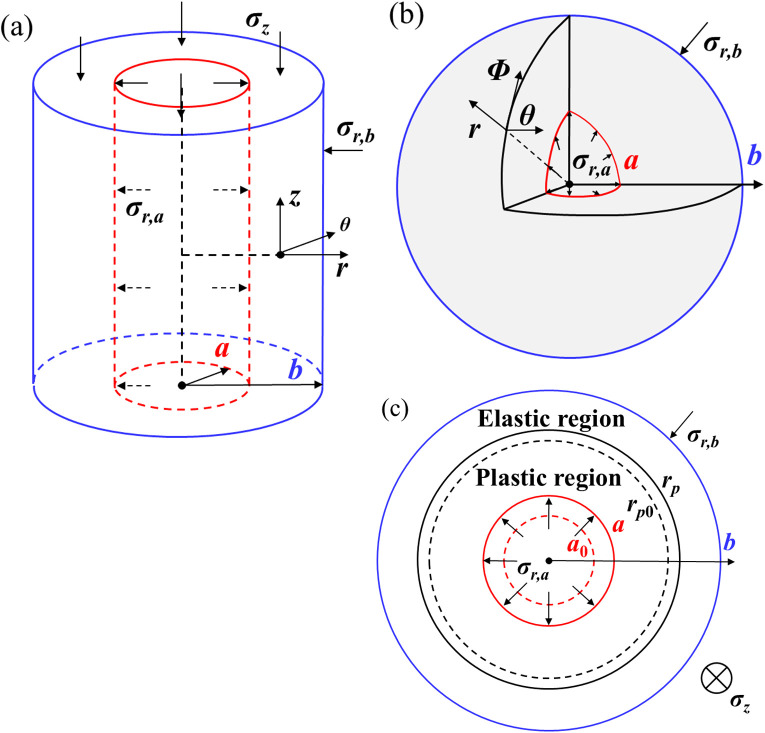
Schematic diagram of a hollow cavity and plane view. (a) Hollow cylinder; (b) Hollow sphere; (c) Plane view for hollow cylinder.

The expansion model of cavity in a finite medium is shown in Fig 1, assuming that the soil is a homogeneous and isotropic material. Adopting cylindrical coordinate system (r, θ, and z) and spherical coordinate system (r, θ, and φ) to describe the positions of elements around the cylindrical or spherical cavity, respectively. The initial radius of the cavity is $a_{0}$, and the radius of the outer boundary is $b_{0}$, and the initial stress inside and outside the cylinder (sphere) is $\sigma_{r0}$. As the pressure on the inner wall of the cavity continues to increase, the radius of the cavity wall expands from $a_{0}$ to a, and the pressure at the cavity wall changes from $\sigma_{r0}$ to $\sigma_{r,a}$. When the boundary conditions of the pressure at the outer wall remain unchanged, the outer radius of the cavity expands from $b_{0}$ to b and the corresponding stress changes from $\sigma_{r0}$ to $\sigma_{r,b}$. The soil around the cavity deforms elastically at the initial loading. As the cavity continues to expand, the soil at the cavity wall will first yield, and then the plastic region expands from the radius of $r_{p0}$ to $r_{p}$. During the expansion process of the soil element around the cavity, the radial ($\sigma_{r}$), circumferential ($\sigma_{\theta }$), vertical ($\sigma_{z}$) (for cylindrical cavity) develop, as well as the corresponding strains ($\varepsilon_{r}$, $\varepsilon_{\theta }$, and $\varepsilon_{z}$).

According to the above description, the stress boundary conditions for the expansion problem of finite medium cylindrical (spherical) cavity are:


$${\;\sigma_{r}|}_{r=a}=\sigma_{r,a}$$ (7a)

$${\;\sigma_{r}|}_{r=b}=\sigma_{r,b}=\sigma_{r,0}$$ (7b)

where r is the radial location of the soil element.

The stress equilibrium equation in the radial direction can be expressed as:


$$\frac{\text{d}\sigma_{r}}{\text{d}r}+k\frac{\sigma_{r}-\sigma_{\theta }}{r}=0$$ (8)

where ‘d(·)’ represents the derivative of a physical quantity with respect to space (Eulerian description); k=1 and 2 represent Eq (8) for cylindrical or spherical cavity analysis, respectively.

The radial displacement ($u_{r}$) of the soil element is given as:


$$u_{r}=r-r_{0}$$ (9)

where r and $r_{0}$ are current and initial locations of the soil element.

During the pure elastic process, the deformation of the soil is small, thus, the deformation in the elastic region is assumed small [21–23], and the strain in the elastic process can be expressed as:


$$\varepsilon_{r}=-\text{d}u_{r}/\text{d}r$$ (10a)

$$\varepsilon_{\theta }=-u_{r}/r$$ (10b)

To accommodate the large deformation in the plastic region, the natural strain is adopted and the strains are given in the natural logarithmic form, as:


$$\varepsilon_{r}=\text{- ln}(dr/\text{d}r_{0})$$ (11a)

$$\varepsilon_{\theta }=\text{- ln}(r/r_{0})$$ (11b)

$$\varepsilon_{v}=\text{- ln}(v/v_{0})$$ (11c)

where v and $v_{0}$ are current and initial volume of the soil element, respectively. For the expansion process of cylindrical cavities, since the horizontal thin disk can be regarded as the plane strain condition, the vertical strain vanishes everywhere, that is, $\varepsilon_{z}=0$.

### 3.2 Solution to the elastic region

The stress-strain relationship of the soil element in the elastic region conform to the Hooke’s law, which can be given for the cylindrical and spherical cavity, respectively, as:


$$\left[\begin{array}{c} \text{D}\varepsilon_{1}^{\text{e}}\\ \text{D}\varepsilon_{2}^{\text{e}}\\ \text{D}\varepsilon_{3}^{\text{e}} \end{array}]=\frac{1}{E}[\begin{array}{ccc} 1 & -\mu & -\mu \\ -\mu & 1 & -\mu \\ -\mu & -\mu & 1 \end{array}][\begin{array}{c} \text{D}\sigma_{1}\\ \text{D}\sigma_{2}\\ \text{D}\sigma_{3} \end{array}\right]$$ (12a)

$$\left[\begin{array}{c} \text{D}\varepsilon_{1}^{\text{e}}\\ \text{D}\varepsilon_{3}^{\text{e}} \end{array}]=\frac{1}{E}[\begin{array}{cc} 1 & -2\mu \\ -\mu & 1-\mu \end{array}][\begin{array}{c} \text{D}\sigma_{1}\\ \text{D}\sigma_{3} \end{array}\right]$$ (12b)

where ‘D(·)’ represents the derivative of a physical quantity with respect to time (Lagrangian description); The superscript ‘e’ represents the physical quantity of the elastic process; μ is Poisson’s ratio.

The stress distribution in the elastic region can be expressed as:


$$\sigma_{r}=\sigma_{r,0}+B_{p}[{\left(b/r\right)}^{k+1}-1]$$ (13a)

$$\sigma_{\theta }=\sigma_{r,0}-B_{p}[{\left(b/r\right)}^{k+1}/k+1]$$ (13b)

where $B_{p}$ can be given with equation (12a) and (12b) when $r=r_{p}$, as:


$$B_{p}=\frac{\sigma_{rp}-\sigma_{0}}{{\left(b/r_{p}\right)}^{k+1}-1}$$ (14)

where $\sigma_{rp}$ is the radial stress at the elastoplastic boundary. When considering $K_{0}$-consolidation, the vertical stress ($\sigma_{z}$) can be give as $\sigma_{z}=K_{0}\sigma_{r,0}-2\mu B_{p}$, where $K_{0}$ is the in-situ coefficient of the earth pressure.

From Equations (12), (14), and (15), and the time process is related to the elastoplastic boundary, the displacement of the soil element within the elastic region can be calculated as:


$$\text{D}u_{r}=\frac{r}{E}\left\{{1-k\mu -2(2-k)\mu^{2}+\frac{1+\mu }{k}\left(\frac{b}{r}\right)}^{k+1}\right\}\text{D}B_{p}$$ (15)

The mean stress (p) can be expressed as $p=(\sigma_{r}+\sigma_{\theta }+\sigma_{z})/3$ for cylindrical cavity expansion process and $p=(\sigma_{r}+2\sigma_{\theta })/3$ for spherical cavity expansion process. Then the mean stress (p) can be given from equations (12a) and (12b), as:


$$p=p_{0}-\frac{k+1-2(k-2)\mu }{3}B_{p}$$ (16)

where $p_{0}$ is the initial mean stress.

Combining Equations (14) and (15), the displacement distribution in the elastic region can be given in the integral form, as:


$$\frac{u_{r}(r)}{r}=-3\int_{p_{0}}^{p_{p}}\frac{{1-k\mu -2(2-k)\mu^{2}+\frac{1+\mu }{k}\left(\frac{b}{r}\right)}^{k+1}}{E[k+1-2(k-2)\mu ]}\text{D}p$$ (17)

Since the mean stress (p) remains unchanged within the elastic region, Eq. (17) can be expressed as:


$$u_{r}=-\frac{3r\left[{1-k\mu -2(2-k)\mu^{2}+\frac{1+\mu }{k}\left(\frac{b}{r}\right)}^{k+1}\right]}{E[k+1-2(k-2)\mu ]}$$ (18)

### 3.3 Solution to the plastic region

The total strain incremental of the soil (Dε) can be decomposed into its elastic ($\text{D}\varepsilon^{\text{e}}$) and plastic ($\text{D}\varepsilon^{\text{p}}$)components. The elastic component is represented by the Hooke’s law from Eqs (12a) and (12b), while the plastic component is modelled using the plastic flow rule of the SDMC model, which can be expressed as follows:


$$\text{D}\varepsilon^{\text{p}}=\frac{1}{K_{p}}\frac{\partial f}{\partial \sigma }\text{:D}\sigma \frac{\partial g}{\partial \sigma }$$ (19)

where $\frac{\partial f}{\partial \sigma }$ and $\frac{\partial g}{\partial \sigma }$ are vectors normal to the yield surface and plastic potential surface. The plastic potential surface of the SDMC model is defined as:


$$g=\sigma_{1}-\alpha_{d}\sigma_{3}$$ (20)

where $\alpha_{d}=(1+\text{sin}\phi_{d})/(1-\text{sin}\phi_{d})$.

In the general space, $\frac{\partial g}{\partial \sigma }$ can be decomposed into maximum, intermediate, and minimum directions, given as:


$$\frac{\partial g}{\partial \sigma_{1}}=1-\frac{\partial \alpha_{d}}{\partial \sigma_{1}}\sigma_{3}=1+g_{s}\sigma_{3}$$ (20a)

$$\frac{\partial g}{\partial \sigma_{2}}=-\frac{\partial \alpha_{d}}{\partial \sigma_{1}}\sigma_{3}=g_{s}\sigma_{3}$$ (20b)

$$\frac{\partial g}{\partial \sigma_{3}}=-\frac{\partial \alpha_{d}}{\partial \sigma_{3}}\sigma_{3}-\alpha_{d}=-\alpha_{d}+g_{s}(p)\sigma_{3}$$ (20c)

where $\sigma_{2}$ is the intermediate tress; $g_{s}(p)$ is given as:


$$g_{s}=\frac{2n^{d}\lambda \zeta cos\psi }{3(1-\text{sin}\psi {)}^{2}\left(1+{n^{d}}^{2}\phi_{d}^{2}\right)p}{\left(\frac{p}{p_{atm}}\right)}^{\zeta }$$ (21)

Similarity, $\frac{\partial f}{\partial \sigma }$ can also be decomposed into maximum, intermediate, and minimum directions, given as:


$$\frac{\partial f}{\partial \sigma_{1}}=1-\frac{\partial \alpha }{\partial \sigma_{1}}\sigma_{3}-\frac{\partial Y}{\partial \sigma_{1}}=1-f_{s}\sigma_{3}\text{ - }Y_{s}$$ (22a)

$$\frac{\partial f}{\partial \sigma_{2}}=-\frac{\partial \alpha }{\partial \sigma_{2}}\sigma_{3}-\frac{\partial Y}{\partial \sigma_{2}}=-f_{s}\sigma_{3}-Y_{s}$$ (22b)

$$\frac{\partial f}{\partial \sigma_{3}}=-\left[\frac{\partial \alpha }{\partial \sigma_{1}}\sigma_{3}+\alpha +\frac{\partial Y}{\partial \sigma_{3}}\right]=-f_{s}\sigma_{3}-\alpha -Y_{s}$$ (22c)

where $f_{s}$ and $Y_{s}$ are given as:


$$f_{s}\text{ = }\frac{-\text{2}n^{b}\lambda \xi \text{cos}\phi \text{tan}\phi_{c}exp(-n^{b}\psi )}{3{\left(1-\text{sin}\phi \right)}^{2}\{1+{\left[\text{tan}\phi_{c}exp(-n^{b}\psi )\right]}^{2}\}p}{\left(\frac{p}{p_{\text{atm}}}\right)}^{\xi }$$ (23a)

$$Y_{s}\text{ = }\frac{-2cn^{b}\lambda \xi \text{tan}\phi_{c}exp(-n^{b}\psi )}{3(1-\text{sin}\phi )\left\{1+{\left[\text{tan}\phi_{c}exp(-n^{b}\psi )\right]}^{2}\right\}p}{\left(\frac{p}{p_{\text{atm}}}\right)}^{\xi }$$ (23b)

Since there is no internal variable in the SDMC model, the plastic modulus should not exist. The stresses and strains of soil elements can be related by stiffness matrix, as:


$$\left[\begin{array}{c} \text{D}\sigma_{1}\\ \text{D}\sigma_{2}\\ \text{D}\sigma_{3} \end{array}]=\frac{1}{H}[\begin{array}{ccc} H_{11} & H_{12} & H_{13}\\ H_{21} & H_{22} & H_{23}\\ H_{31} & H_{32} & H_{33} \end{array}][\begin{array}{c} \text{D}\varepsilon_{1}\\ \text{D}\varepsilon_{2}\\ \text{D}\varepsilon_{3} \end{array}\right]$$ (24)

where $H_{ij}$ is matrix elements, given as:


$$H=E(\mu +1)\left[(1-\mu )\left(A_{1}B_{1}+A_{2}B_{2}+A_{3}B_{3}\right)+\mu (A_{1}B_{2}+A_{2}B_{1}+A_{1}B_{3}+A_{3}B_{1}+A_{2}B_{3}+A_{3}B_{2})\right]$$ (25a)

$$H_{ii}=E^{2}\left[\left(A_{j}B_{j}+A_{k}B_{k}\right)+\mu \left(A_{j}B_{k}+A_{k}B_{j}\right)\right]\;(i,j,k=1,2,3;i\neq j\neq k)$$ (25b)

$$H_{ij}=E^{2}\left[-A_{i}B_{j}+\mu \left(A_{k}B_{k}-A_{i}B_{k}-A_{k}B_{j}\right)\right]\;(i,j,k=1,2,3;i\neq j\neq k)$$ (25c)

and


$$A_{i}=\frac{\partial g}{\partial \sigma_{i}}\;(i=1,\;2,\;3)$$ (26a)

$$B_{i}=\frac{\partial f}{\partial \sigma_{i}}\;(i=1,\;2,\;3)$$ (26b)

Yu et al. [21–23] provided an exact solution for the cavity expansion problem in finite medium using the M-C criterion. Due to the simplicity of the M-C criterion, the Lagrangian method can be used to establish the relationship between stress and total strain, and then obtain the analytical solution. This paper adopts the SDMC model, which believes that the state of soil elements is related to the void ratio and mean stress of the soil, that is, the void ratio and mean stress are functions related to time and space. At this time, it is difficult to derive analytical solutions, and because the SDMC model is developed based on the M-C model, which is an ideal elastoplastic model, the stress and strain increments in its plastic development process are independent. The present study uses the hybrid Eulerian-Lagrangian (HEL) method [7–9] to solve the cavity expansion problem during the plastic process.

The radial, circumferential, and vertical strains provided in Eqs (11a-c) are not independent and should satisfy $\text{D}\varepsilon_{v}=\text{D}\varepsilon_{r}+\text{D}\varepsilon_{\theta }$ for cylindrical cavity and $\text{D}\varepsilon_{v}=\text{D}\varepsilon_{r}+2\text{D}\varepsilon_{\theta }$ for spherical cavity, thereby giving compatibility equation in terms of r and v as:


$$\text{d}r=\frac{v}{v_{0}}{\left(\frac{r_{0}}{r}\right)}^{k}\text{d}r_{0}\;$$ (27)

Combining Eqs (8) and (27), the stress equilibrium equation can be transformed to be the expression of $\sigma_{r}$ in the Eulerian description as:


$$\text{d}\sigma_{r}=k(\sigma_{\theta }-\sigma_{r})\frac{v}{v_{0}}{\left(\frac{r_{0}}{r}\right)}^{k}\text{d}r_{0}$$ (28)

Eq (24) can also be expressed reversely by the flexibility matrix, as:


$$\left[\begin{array}{c} \text{D}\varepsilon_{1}\\ \text{D}\varepsilon_{2}\\ \text{D}\varepsilon_{3} \end{array}]=[\begin{array}{ccc} A_{11} & A_{12} & A_{13}\\ A_{21} & A_{22} & A_{23}\\ A_{31} & A_{32} & A_{33} \end{array}][\begin{array}{c} \text{D}\sigma_{1}\\ \text{D}\sigma_{2}\\ \text{D}\sigma_{3} \end{array}\right]$$ (29)

where the matrix [A] can be calculated by the inverse of the matrix [H]/H of Eq (24).

Then, the magnitude of radial, circumferential, and vertical stresses (for cylindrical cavity) should be compared at this time. For example, for a soil element within the plastic region around a cylindrical cavity, when the maximum, intermediate, and minimum stresses correspond to radial, vertical, and circumferential stresses, respectively, i.e. $\sigma_{1}=\sigma_{r}$, $\sigma_{2}=\sigma_{z}$, and $\sigma_{3}=\sigma_{\theta }$, substituting Eqs (11b-c) into Eq (29), the hoop stress, vertical stress, and specific volume can be expressed in the incremental form, as:


$$\left[\begin{array}{c} \text{D}\sigma_{z}\\ \text{D}\sigma_{\theta } \end{array}]=[\begin{array}{cc} A_{\theta \theta } & A_{\theta z}\\ A_{z\theta } & A_{zz} \end{array}]-1[\begin{array}{c} -A_{\theta r}\text{D}\sigma_{r}-\text{D}r/r\\ -A_{zr}\text{D}\sigma_{r} \end{array}\right]$$ (30a)

$$\frac{\text{D}v}{v}=-[\begin{array}{c} A_{rr}+A_{\theta r}+A_{zr}\\ A_{r\theta }+A_{\theta \theta }+A_{z\theta }\\ A_{rz}+A_{\theta z}+A_{zz} \end{array}]\text{T}[\begin{array}{c} \text{D}\sigma_{r}\\ \text{D}\sigma_{\theta }\\ \text{D}\sigma_{z} \end{array}]$$ (30b)

By comparing the magnitude of radial, circumferential, and vertical stresses (for cylindrical cavity), different results of Eq (30a) and (30b) can be obtained. Now five PDEs for the analysis in the elastoplastic zone have been obtained by the combination use of Eulerian description from Eqs (27) and (28) and Lagrangian description from Eqs (30a) and (30b). Consequently, stresses and strains in the plastic region can be calculated with the information at the elastoplastic boundary. However, both the material time derivative (Lagrangian description) and the spatial derivative (Eulerian description) are involved in the governing PDEs for the elastoplastic cavity expansion analysis, and they cannot be transformed into ordinary differential equations (ODEs). With the HEL approach, the cylinder (sphere) of soil is discretized into (m−1) concentric annuli, where m represents the number of nodes. Simultaneously, the loading process is divided into a number of continuous load steps. Each node is marked by its initial position as ${r\prime }_{\left(i\right)}^{\left(0\right)}$, where the subscript i=1, 2, 3,…, m denotes the ith node and the superscript denotes the number of load step. The distribution of nodes is set to follow the nonlinear function in this study:


$$r_{\left(i\right)}^{\left(0\right)}={\left(\frac{b}{a}\right)}^{\frac{1}{m-1}}r_{\left(i\right)}^{\left(0\right)}$$ (31)

An information vector ($x_{\left(i\right)}^{\left(j\right)}$) can be defined for the ith node at the jth load step, including equivalent radial location, stresses, and deformation conditions, as:


$$x_{\left(i\right)}^{\left(j\right)}={\left[r_{\left(i\right)}^{\left(j\right)},\sigma_{r(i)}^{\left(j\right)},\sigma_{\theta (i)}^{\left(j\right)},\sigma_{z(i)}^{\left(j\right)},v_{\left(i\right)}^{\left(j\right)}\right]}^{\text{T}}$$ (32)

where the superscript “j” represents the jth load step.

At the jth step, the increment of x from node (i+1) to node (i) is defined as:


$$\text{d}x=x_{\left(i\right)}^{\left(j\right)}-x_{\left(i+1\right)}^{\left(j\right)}$$ (33)

Similarly, for the ith node upon loading from the load step (j−1) to load step (j), Dx equals to:


$$\text{D}x=x_{\left(i\right)}^{\left(j\right)}-x_{\left(i\right)}^{\left(j-1\right)}$$ (34)

where $x_{\left(i\right)}^{\left(j-1\right)}$ is known from the previous step of loading. Having calculated $r_{\left(i\right)}^{\left(j\right)}$ and $\sigma_{r(i)}^{\left(j\right)}$ from Eqs (27), (28), and (33), $\text{D}r_{\left(i\right)}^{\left(j\right)}$ and $\text{D}\sigma_{r(i)}^{\left(j\right)}$ can be known by Eq (34). Then, $\text{D}\sigma_{\theta }$, $\text{D}\sigma_{z}$, and Dv can be determined from Eqs (30a) and (30b) with $x=x_{\left(i\right)}^{\left(j-1\right)}$, and $\sigma_{\theta (i)}^{\left(j\right)}$, $\sigma_{z(i)}^{\left(j\right)}$, and $v_{\left(i\right)}^{\left(j\right)}$ can also be calculated. The detailed solving procedure can refer to Yang et at. [7–9].

The Initial value for the solving procedure of the HEL approach is the same as the soil state at the elastoplastic boundary, derived from Eqs (13a) and (13b) as:


$$\sigma_{rp}=\sigma_{r,0}+B_{p}[{\left(b/r_{p}\right)}^{k+1}-1]$$ (35a)

$$\sigma_{\theta p}=\sigma_{r,0}-B_{p}[{\left(b/r_{p}\right)}^{k+1}/k+1]$$ (35b)

and $B_{p}$ can be given as:


$$B_{p}=\frac{Y+(\alpha -1)\sigma_{r,0}}{(\alpha +1)\left[{\left(\frac{b}{r_{p(1)}^{\left(j\right)}}\right)}^{2}+(\alpha +1)\right]}$$ (36)

where $\sigma_{\theta p}$ is the circumferential stress at the elastoplastic boundary and $\sigma_{zp}=\sigma_{r,0}$ can be given for the vertical stress at the elastoplastic boundary for the cylindrical cavity.

**Fig 2:**
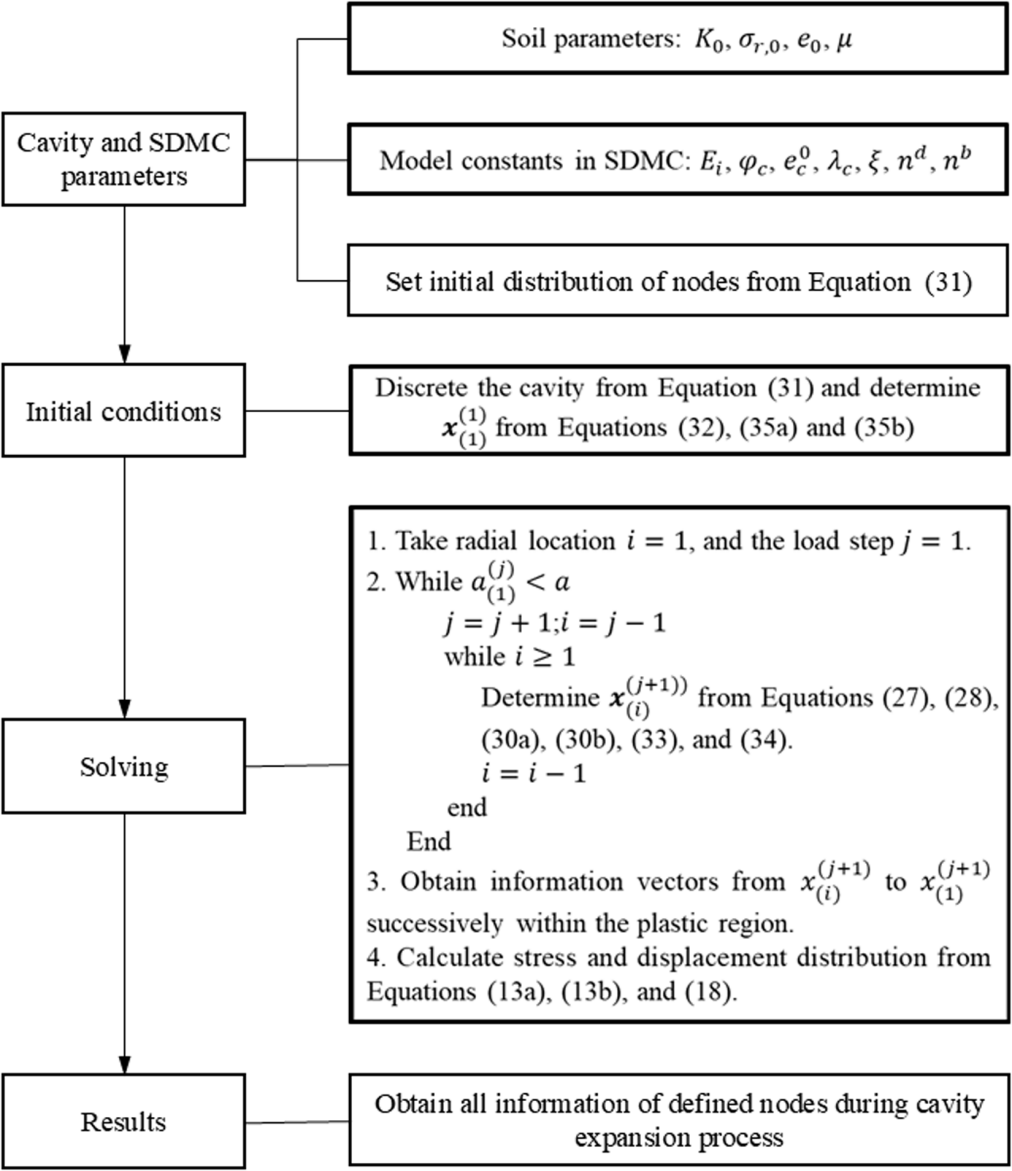
Solving procedure for the solution of a cavity expansion process.

Finally, the solving procedure for the solution of a cavity expansion process is detailed as Fig 2.

## 4. Validation

To verify the proposed approach, finite-element (FE) simulations were conducted by software Plaxis2d and Abaqus. The FE simulation established in the Plaxis2d was used to validate the conversation of principal stress during cylindrical cavity expansion, because the classical M-C model is incorporated in the software. Then, the accuracy of the present approach with the SDMC model in this paper was verified with the model established in the Abaqus, with the M-C model which was user-modified as the SDMC model. Since the form of the M-C model is adopted as the William form [24], of which the angle of the yield surface of the traditional M-C model is rounded, the validation of conversion of the principal stress and the accuracy of the proposed method can be roughly conducted. Finally, an exist exact solution is compared to verify the accuracy of the presented solving procedure of HEL method with M-C model.

### 4.1 Validation for the conversation of principal stresses

**Fig 3:**
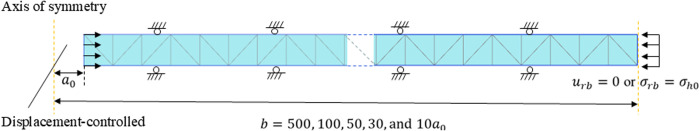
Meshing model established in Plaxis2d.

Since the cavity expansion problem was symmetrical, the simulation model was established using the axisymmetric shell elements, as shown in Fig 3. The soil was modelled as the M-C soil and the soil’s parameters were shown in Table 1, and other parameters needed to be set in Plaxis2d were adopted as default values. The initial cavity radius is set as $a_{0}$, with the outer boundary of $b=500a_{0}$, which was far enough to eliminate the boundary effect. Normal displacements of upper and lower boundaries are restricted, and a constant normal stress of $\sigma_{r,0}$ was applied to the outer boundary. The model was meshed using the triangular elements and the grid refinement was enhanced. The quality of the element distribution was medium and the number of the elements were 1066 at last.

**Table 1:** Soil characteristics in the simulation model of Plaxis2d.

Parameters	Values
Initial void ratio $e_{0}$	0.63
Elastic modulus E (MPa)	70
Cohesion force c (kPa)	0
Friction angle ϕ (∘)	33
Dilation angle $\phi_{d}$ (∘)	3

There were three steps including in the simulation. First, all directions of boundaries were restricted. Since the plane-strain condition was adopted, the calculation mode was set as field stress with $\sigma_{r,0}=67.308\;\text{kPa}$ and $\sigma_{z,0}=158\;\text{kPa}$ and 67.308 kPa corresponding to $K_{0}=\sigma_{r,0}/\sigma_{z,0}=0.426$ and 1, respectively. Secondly, a plastic calculation was added with no variation to avoid imbalance of the model. Thirdly, the restrictions of the upper and lower boundaries were changed to the normal direction. The restrictions of inner and outer boundaries were released, and the inner wall moved radially to the location of $a=1.11a_{0}$ with a constant stress of $\sigma_{r,0}=67.308\;\text{kPa}$ applied to the outer boundary.

**Fig 4:**
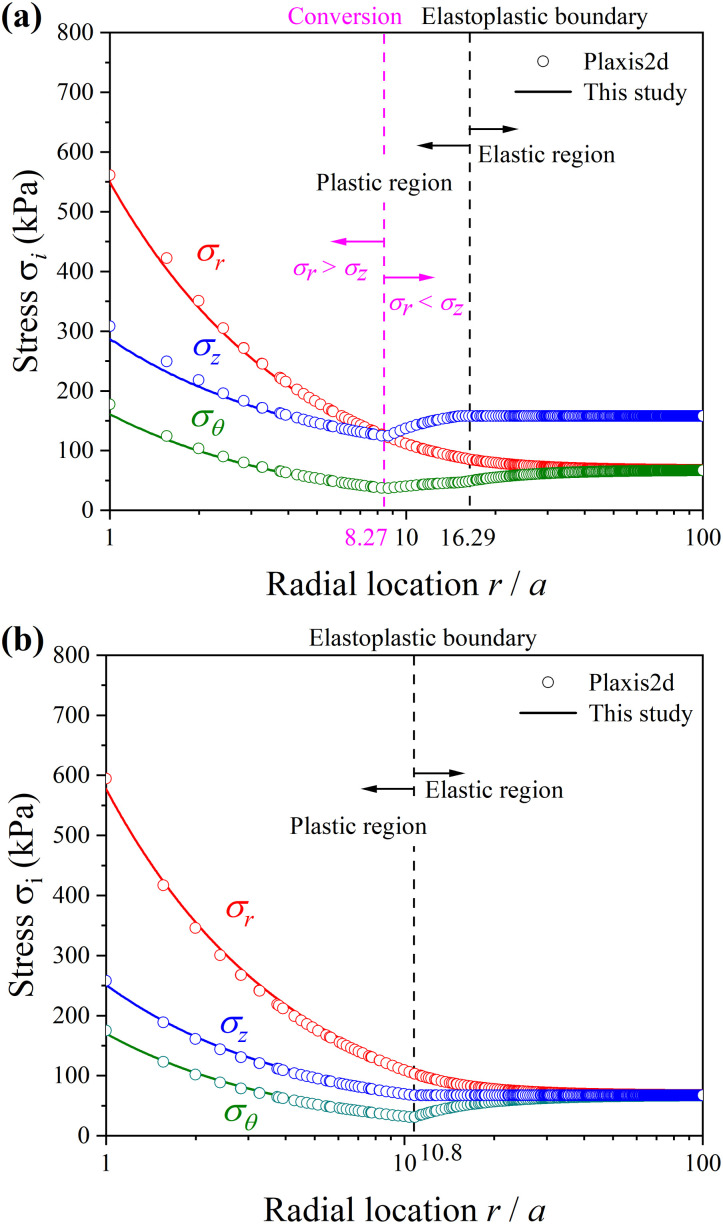
Comparison of results of stress distributions between present study and numerical simulation from Plaxis2d. (a) $K_{0}=0.426$; (b) $K_{0}=1$.

The solving procedure based on the SDMC model in study is degraded to accommodate to that based on the M-C model. The comparison of results between present study and numerical simulation from Plaxis2d is shown in Fig 4, which shows good agreements. Since the cavity expansion process in infinite medium is self-similar, the distribution of stresses reflects the stress paths of soil elements around the cavity. Fig 4(a) shows the conversion of principal stresses during cavity expansion process when $K_{0}=0.426$. The radial ($\sigma_{r}$) stress equals to the circumferential stress ($\sigma_{\theta }$), and both stresses are smaller than the vertical stress ($\sigma_{z}$) of the soil elements at initial loading. During elastic loading, $\sigma_{z}$ remains constant while $\sigma_{r}$ and $\sigma_{\theta }$ develop. When the soil yields, $\sigma_{z}$ and $\sigma_{\theta }$ become smaller, and $\sigma_{r}$ continues increasing until $\sigma_{z}=\sigma_{r}$. At this time, the maximum stress changes from $\sigma_{z}$ to $\sigma_{r}$, then all stresses increase with the loading increases. Both present solutions and Plaxis2d results capture the conversion of principal stresses. However, this phenomenon of the conversion of principal stresses can not be seen for the cavity expansion process when $K_{0}=1$. The value of radial stress ($\sigma_{r}$) has already been larger than that of the vertical stress ($\sigma_{z}$) when the soil yields. Thus, all stresses increase, and the maximum, intermediate, and minimum stresses are radial, vertical, and circumferential stresses, respectively, that is, $\sigma_{1}=\sigma_{r}$, $\sigma_{2}=\sigma_{z}$, and $\sigma_{3}=\sigma_{\theta }\;$ all the time.

### 4.2 Validation for the cavity expansion with the SDMC model

Different from the classical M-C model in plaxis2d, the M-C model adopted in Abaqus adopts the form of the William Mohr-Coulomb model, of which the detailed equations are presented in the Appendix [24]. The parameters of soil characteristics and constants in the SDMC model take from NE34 sand provided by Ye et al. [25], as presented in Table 2.The state parameter (ψ) is taken within the range of [−0.5,0.5] to fit the friction angle (ϕ) and the dilation angle ($\phi_{d}$) in the linear form as ϕ=−55.23ψ+34.89 and $\phi_{d}=-86.31\psi$.

**Table 2:** Soil characteristics and constants in the SDMC model for the NE34 sand [25].

Elastic parameters	Critical state parameters	State-dependent parameters
$E_{i}\text{ = 250}$	$\phi_{c}=30\circ$	$n_{d}\text{ = 1.7}$
μ = 0.3	$e_{c\text{0}}\text{ = 0.9}$	$n_{b}\text{ = 2.3}$
	$\lambda_{c}\text{ = 0.119}$	
	ξ = 0.23	

**Fig 5:**
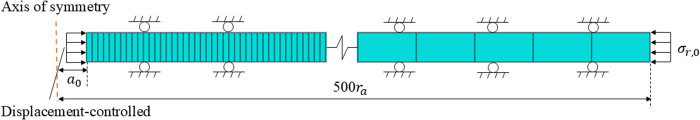
Meshing model established in Abaqus.

The model establishment methods provided by Zhou et al. [26] and developed by Pang et al. [27] are adopted in this study. The meshing model is depicted Fig 5. The initial radius ($a_{0}$) of the cavity wall is assumed to be 1, and the boundary effects are eliminated by adopting a sufficient length of the outer boundary with 500$r_{a}$. The size of the element decreases near the cavity wall to ensure the accuracy of results of numerical simulation. Then, the element number of the model is 5360 with eight-node axisymmetric reduced-integration (CAX8R) elements, and the void ratio field is defined with $e_{0}=0.63$.

Three steps are included in the numerical simulation of Abaqus. First, the initial stress field is defined in the entire soil through the “predefined field” in ABAQUS of $\sigma_{v0}=158\;\text{kPa}$ and $\sigma_{h0}=67.308\;\text{kPa}$, with $K_{0}=\sigma_{h0}/\sigma_{v0}=0.426$. The other initial stress field with $\sigma_{v0}=37.308\;\text{kPa}$ and $K_{0}=1$ is also defined to analyze the effect of the in-situ coefficient of the earth pressure ($K_{0}$), the same as those defined in Plaxis2d. In this way, the initial mean stress ($p_{0}$) is calculated as 146.8 kPa and 67.308 kPa for $K_{0}=0.426$ and 1.0, respectively. Additionally, some keywords should be added in ABAQUS as $\phi_{0}=-0.1404$ and $E_{0}=30443\;\text{kPa}$ for $K_{0}=0.426$ and $\phi_{0}=-0.1635$ and $E_{0}=19835\;\text{kPa}$ for $K_{0}=1$ to provide the initial values of ϕ and E. Second, upper and lower boundaries of the cavity wall are restricted by fixing the displacement of the normal direction, while a radial stress equal to the initial total radial stress of the soil is applied to the outer boundary to achieve a geostatic stress equilibrium. Third, the restriction of the cavity wall is removed, and a displacement-controlled boundary with uniform radial displacement is applied to the cavity wall to simulate the cavity expansion process with $a=1.11a_{0}$.

**Fig 6:**
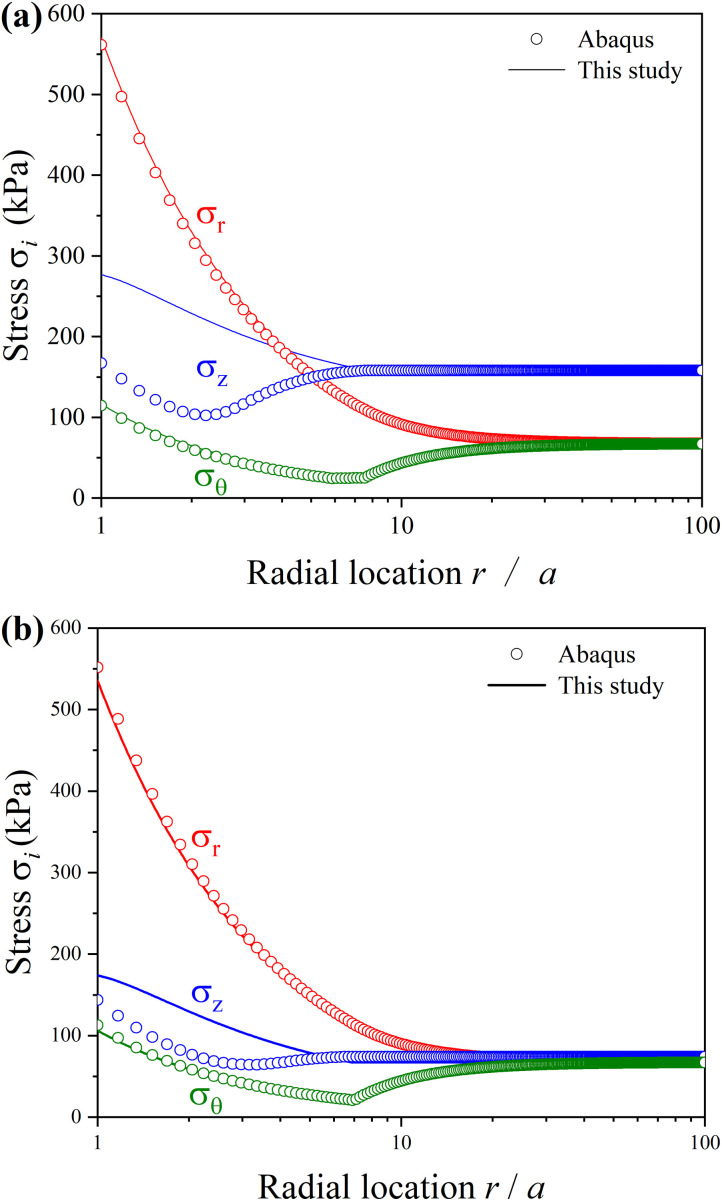
Comparison of stress distributions between results of this study and Abaqus. (a) ${𝐊}_{0}=0.426$; (b) ${𝐊}_{0}=1$.

As depicted in Fig 6, the soil deforms elastically outside the elastoplastic boundary ($r_{p}$) which roughly equals to 8a, and deforms elastoplastically within $r_{p}$. The results of distributions of radial ($\sigma_{r}$) and circumferential ($\sigma_{\theta }$) stresses of the present solution are the same as those from numerical simulation. A significant difference is shown for distributions of the vertical stress ($\sigma_{z}$), because the relationship between the vertical stress ($\sigma_{z}$) and strain ($\varepsilon_{z}$) obeys the user-modified William Mohr-Coulomb in ABAQUS. However, results of the distribution of $\sigma_{r}$ and $\sigma_{\theta }$ are the same as those from numerical simulation, which verifies the accuracy of the present study.

### 4.3 Validation for the limited boundary cases

Yu [19] proposed an exact solution to the cavity expansion in the finite medium with the M-C model, of which the results are compared with those derived from the solution of the present study to verify the used HEL solving procedure. At this time, the elastic modulus (E) of the soil doesn’t change with the mean stress (p), and the friction angle (ϕ) and dilation angle ($\phi_{d}$) remains constant. Then the calculation of specific volume (v) and the distribution of displacement ($u_{r}$) can be simplified as [8]:


$$\frac{v_{0}}{v}=1+\frac{3(1-2\mu )(p-p_{0})}{E}$$ (37a)

$$\frac{u_{r}}{r_{0}}\text{ = }\frac{B_{p}(1+\mu )}{E}[1-\mu +(\frac{b_{0}}{r_{0}}{)}^{2}]$$ (37b)

**Fig 7:**
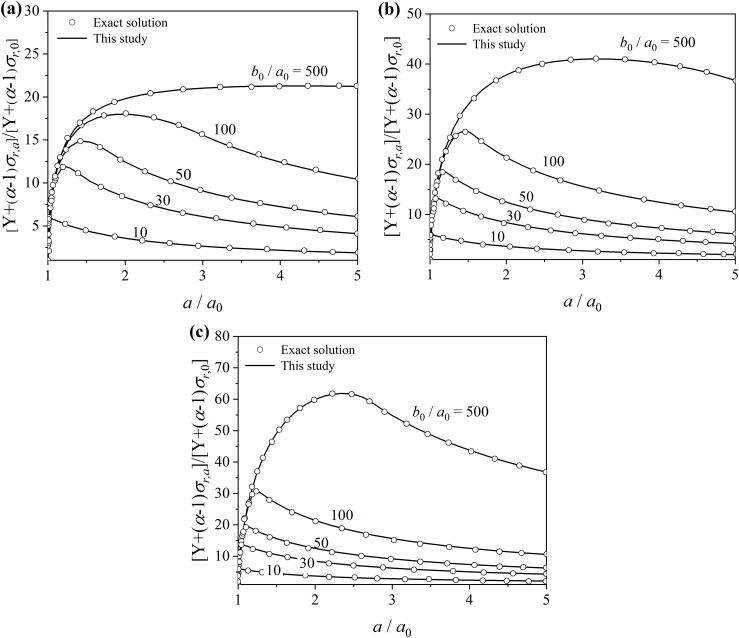
Comparison of results of pressure-expansion curves between the present study and exact solution. (a) $\phi_{d}=0\circ$; (b) $\phi_{d}=20\circ$; (c) $\phi_{d}=40\circ$;.

The Poison’s ratio and friction angle are taken as μ=0.3 and ϕ=40∘, respectively. To ignore the effect of initial stresses ($p_{0}$) and cohesion force (c), Yu [21] adopted a general stiffness index of the cohesion-frictional material, defined as $E/[(\alpha -1)\sigma_{r}+Y]$, and took it as 500. Fig 7 illustrates the comparison of results of pressure-expansion curves between the present study and exact solution. It can be seen the present solution can agree well with the exact solution for different thicknesses of cylinder and dilation angles.

## 5. Application to the CPT

### 5.1 Mini cone penetration test (MCPT)

**Fig 8:**
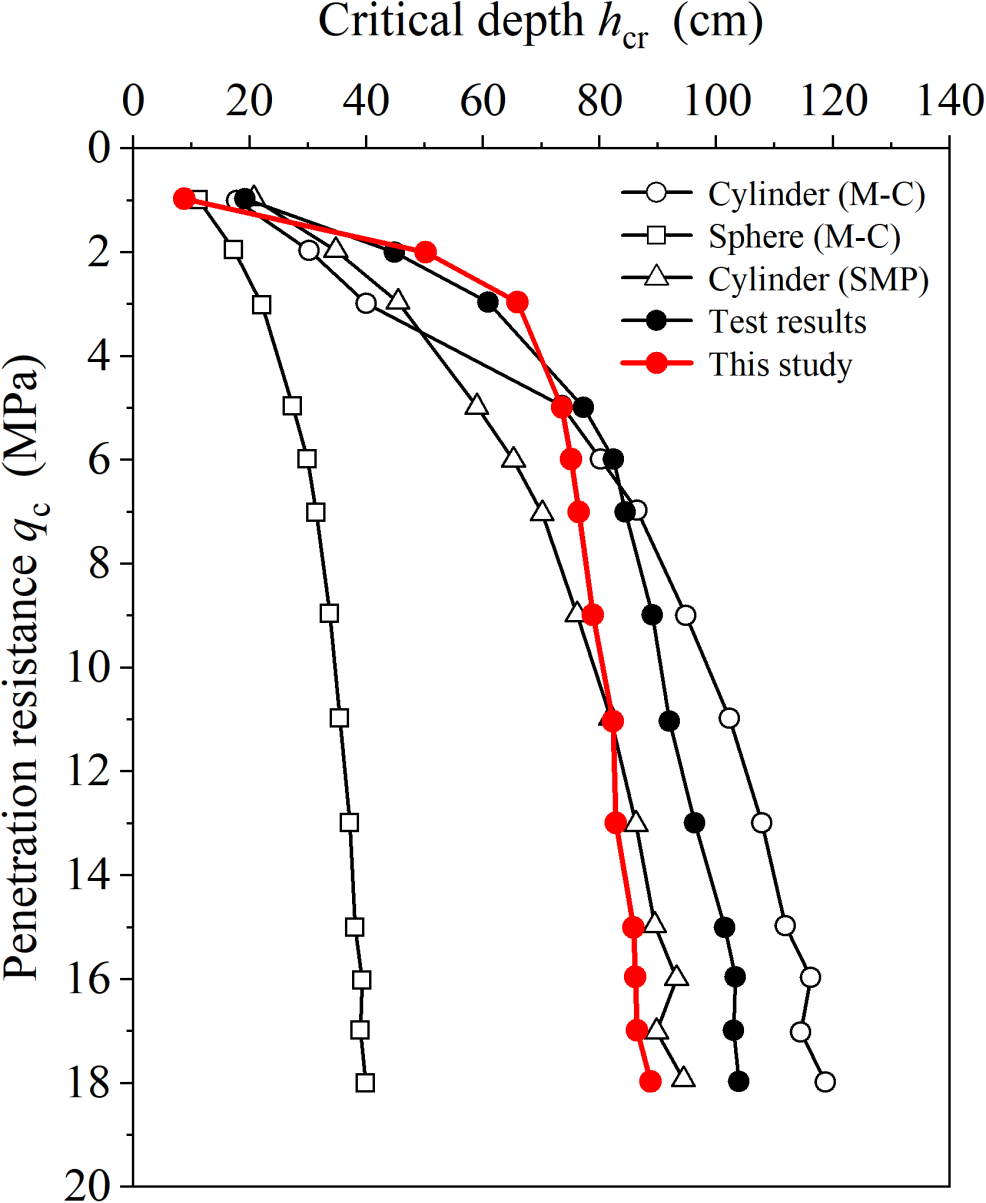
Penetration resistance of MCPT.


The proposed method is first applied to predicting the response of the CPT during penetration. Cui and Ding [28] conducted a series of CPT using a micro penetrometer (MCPT). Fig 8 shows the penetration curve of a MCPT with a cone size of 12 mm in soil with a density of 95%. The concept of the critical depth ($h_{cr}$) was proposed by Kerisel [29], which means the resistance of the cone will no longer increases or increases slowly when penetrating to a certain depth. $h_{cr}$ can be calculated using the semi-empirical equation for the critical depth of ultimate penetration resistance proposed by Li et al. [30], as follows:

$$h_{\text{cr}}=F(\phi )\cdot (2a{)}^{\frac{2\text{sin}\phi }{1+3\text{sin}\phi }}\cdot {\left(\frac{q_{c}}{\gamma }\right)}^{\frac{1+\text{sin}\phi }{1+3\text{sin}\phi }}$$ (38)

where F(ϕ) is the function of ϕ; γ is the soil weight; $q_{c}$ is the penetration resistance.

The boundary effect is ignored in this comparison. The values of parameters for $\phi_{c}$ and γ are provided in Table 3. $a/a_{0}=5$ can be assumed and the Poison’s ratio (μ) and modulus (E) of the soil are taken as 0.3 and 54 MPa, respectively, the other constants in the SDMC model are shown in Table 4 provided by Gao et al. [31].

**Table 3:** Parameters for the soil [26].

$\phi_{c}(\circ )$	33	34	34.5	35	36	37	38	39	40
$\gamma (g\text{/}{cm}^{\text{3}})$	1.55	1.6	1.6	1.6	1.65	1.65	1.66	1.67	1.67

**Table 4:** Constants in the SDMC model [31].

$e_{c\text{0}}$	$\lambda_{c}$	ξ	$n^{d}$	$n^{b}$
0.9	0.119	0.23	1.53	2.68

Fig 8 shows that when penetration resistance ($q_{c}$) is low, the results of the critical depth ($h_{cr}$) derived from the proposed solution can agree well with the MCPT. However, as the penetration resistance ($q_{c}$) increases, the results of the present solution gradually tend to stabilize, which is consistent with the results in Fig 7(a) when $b_{0}/a_{0}$ is large, e.g. $b_{0}/a_{0}=500$, but relatively small compared to the test results. The results of the critical depth derived from the proposed method are larger than those derived from cylindrical and spherical cavity expansion solutions when penetration resistance is small, but are similar to those derived from the spherical cavity expansion when penetration is large.

### 5.2 CPT calibration chamber test

**Fig 9:**
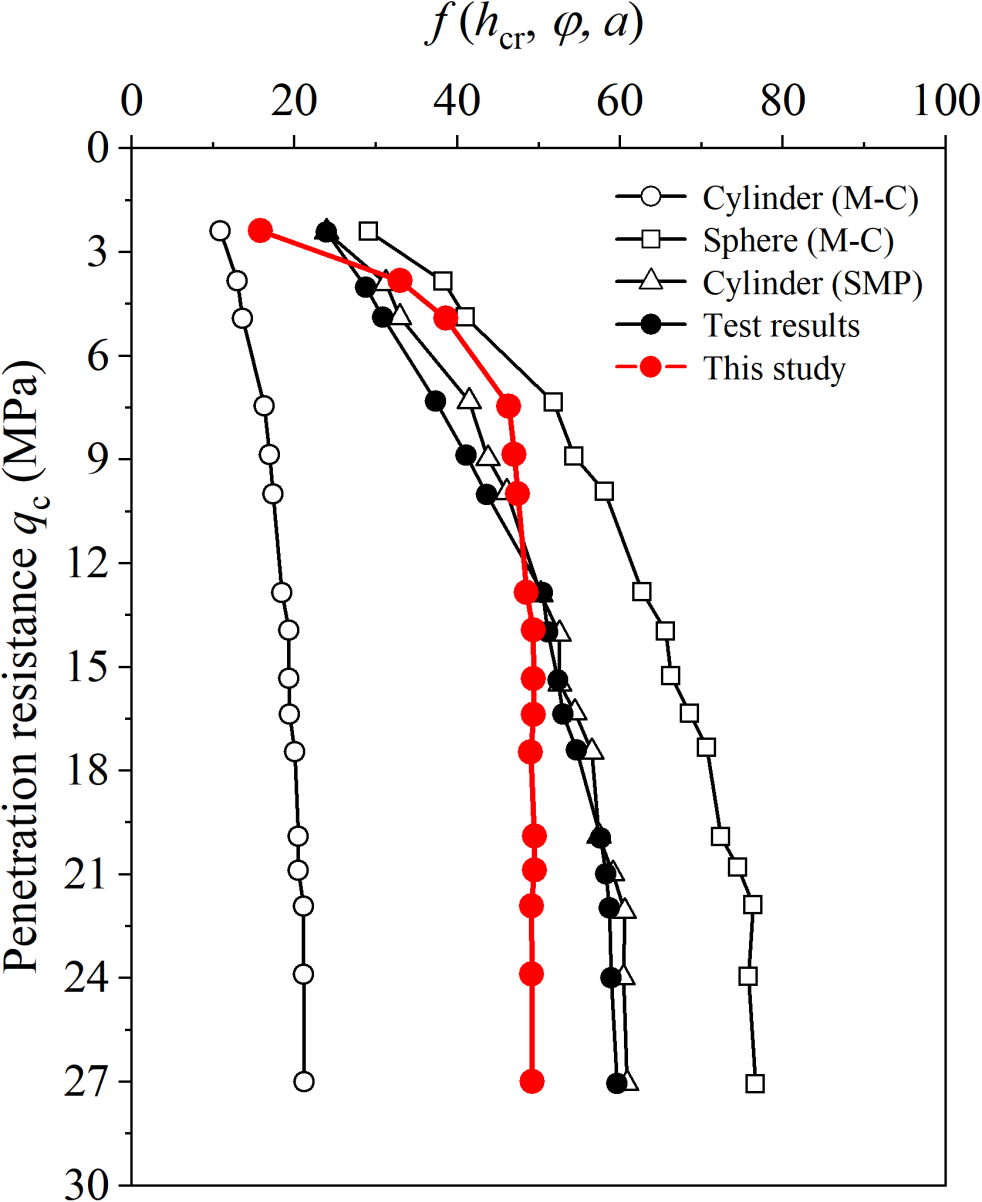
Penetration resistance of CPT calibration chamber test.

Li et al. [30] recalculated the results of the CPT calibration chamber test performed by Kerisel [29] based on the relationship between $h_{cr}\propto q_{c}^{\frac{1+\text{sin}\phi }{1+3\text{sin}\phi }}$, as shown in Fig 9. It is difficult to avoid boundary effects in the calibration chamber test, and the ratio of the outer and inner walls ($b_{0}/a_{0}$) is assumed to be 30. The Poisson’s ratio (μ) is 0.3, and constants in the SDMC model are the same as those provided by Gao et al. [31]. It can be seen that with the increases of penetration resistance, the correlation function of the critical depth $f(h_{cr},\;\phi ,\;a)$ calculated by the proposed method quickly reach a stable value, while $f(h_{cr},\;\phi ,\;a)$ of the test results increase with the increase of penetration resistance. Because of the boundary effect assumed in the present paper, $f(h_{cr},\;\phi ,\;a)$ has a slight decrease when the penetration resistance develops., which is consistent with the results in Fig 7(a) when $b_{0}/a_{0}$ is small, e.g. $b_{0}/a_{0}=30$. Different from the results of the MCPT in Section 5.1, when considering limited boundary, the results of $f(h_{cr},\;\phi ,\;a)$ derived from the proposed method always between those derived from cylindrical and spherical cavity expansion solutions, which are more reasonable and accurate.

## 6. Conclusion

The state-dependent Mohr-Coulomb (SDMC) criterion is adopted in this study to consider the unavoidable boundary effect in CPT calibration chamber test, and derives a semi-analytical solution for cavity expansion in the finite medium. Using the HEL method to solve the stress-strain partial differential equations (PDEs), the stress distribution curves and pressure-expansion curves of the cavity in a finite medium is obtained, and compared with the numerical simulation and exact solutions to verify the accuracy of the method proposed in this paper. Subsequently, the results of the proposed method in this paper are compared with the results of mini cone penetration test (MCPT) and CPT calibration chamber test, and the following conclusions are obtained:

(1) When boundary effects are not considered, the critical depth of ultimate penetration resistance increases with the increase of penetration resistance. However, the results obtained using the proposed method will gradually stabilize, and the critical depth increases rapidly when the penetration resistance is relatively small, which is close to the experimental results.
(2) When considering boundary effects, the critical depth increases rapidly when the penetration resistance is low, while when the penetration resistance is high, the calculated critical depth still increases slowly according to experimental results. However, due to the consideration of boundary effects in this paper, the critical depth tends to stabilize and even slightly decreases when the penetration resistance is high.
(3) The results of cavity expansion using the Mohr-Coulomb (M-C) model are not the same as the experimental results. The solutions by using the cylindrical cavity expansion are relatively large, but those by using the spherical cavity expansion are very small. It can be deduced that the results of CPT are between the results of cylindrical and spherical cavity expansions, and are related to the soil state.

## Appendix A: William Mohr-Coulomb Model

The yield function of the William Mohr-Coulomb model [24] could be written in terms of the stress invariants as:


$$F=R_{mc}q+p\text{tan}\phi -c$$ (A.1)

where $R_{mc}$ is a function of the friction angle (ϕ) and the Lode angel ($\theta_{L}$) given by:


$$R_{mc}=\frac{1}{\sqrt{3}\text{cos}\phi }\text{sin}\left(\theta_{L}+\frac{\pi }{3}\right)+\frac{1}{3}cos(\theta_{L}+\frac{\pi }{3})\text{tan}\phi$$ (A.2)

The plastic potential function for the model is taken as hyperbolic function in the meridional stress plane and a smooth elliptic function in the deviatoric stress plane:


$$G=\sqrt{{\left(\in c\text{tan}\phi_{d}\right)}^{2}+{\left(R_{mw}q\right)}^{2}}+p\text{tan}\phi_{d}$$ (A.3)

where ∈ is a parameter referred to as the meridional eccentricity and taken as 0.1 in the numerical simulation; $R_{mw}$ is calculated by:


$$R_{mw}=\frac{4\left(1-e_{\phi }^{2}\right){\text{cos}}^{2}\theta_{L}+{\left(2e_{\phi }-1\right)}^{2}}{2\left(1-e_{\phi }^{2}\right)\text{cos}\theta_{L}+(2e_{\phi }-1)\sqrt{4\left(1-e_{\phi }^{2}\right){\text{cos}}^{2}\theta_{L}+5e_{\phi }^{2}-4e_{\phi }}}\frac{3-\text{sin}\phi }{6\text{cos}\phi }$$ (A.4)

where $e_{\phi }=(3-sin\phi )/(3+sin\phi )$.

## Supporting information

S1 Data Minimal data set. (ZIP)
