# Supplementary material for: Cavity expansion theory with state-dependent mohr-coulomb model and its application to cone penetration tests
Source: PLoS One. 2025 Aug 20;20(8):e0329935. doi: 10.1371/journal.pone.0329935 (PMC12367164; doi:10.1371/journal.pone.0329935)
Supplement: S1 Data — (ZIP) [file pone.0329935.s001.zip › minimal data set/Data instruction.docx]

All files with the “.obju” suffix should be opened using Origin software.

The file with the “.p2dx” suffix should be opened using Plaxis2d software.

The file with the “.odb” suffix should be opened using Abaqus software.

Files “Fig 4(a)”, “Fig 4(b)”, “Fig 6(a)”, “Fig 6(b)”, “Fig 7(a)”, “Fig 7(b)”, “Fig 7(c)”, “Fig 8” and “Fig 9” contain data and figures corresponding to related figures in the paper.

File “test-Plaxis2d” contains the established finite element model. By performing finite element simulation based on the model, the results can provide data in Figs. 4(a) and (b).

File “test-Abaqus” contains the numerical simulation results incorporating the SDMC model, and provides data in Figs. 6(a) and (b).
